# Clinical Neuropsychology as a Specialist Profession in European Health Care: Developing a Benchmark for Training Standards and Competencies Using the Europsy Model?

**DOI:** 10.3389/fpsyg.2020.559134

**Published:** 2020-10-06

**Authors:** Laura Hokkanen, Fernando Barbosa, Amélie Ponchel, Marios Constantinou, Mary H. Kosmidis, Nataliya Varako, Erich Kasten, Sara Mondini, Sandra Lettner, Gus Baker, Bengt A. Persson, Erik Hessen

**Affiliations:** ^1^Department of Psychology and Logopedics, Faculty of Medicine, University of Helsinki, Helsinki, Finland; ^2^Laboratory of Neuropsychophysiology, Faculty of Psychology and Education Sciences, University of Porto, Porto, Portugal; ^3^GHU Paris Psychiatry & Neuroscience, Paris, France; ^4^Department of Social Sciences, University of Nicosia, Nicosia, Cyprus; ^5^Lab of Cognitive Neuroscience, School of Psychology, Aristotle University of Thessaloniki, Thessaloniki, Greece; ^6^Research Center of Neurology, Lomonosov Moscow State University, Moscow, Russia; ^7^Department of Psychology - Neurosciences, MSH University of Applied Sciences & Medical University, Hamburg, Germany; ^8^Department of Philosophy, Sociology, Education and Applied Psychology, University of Padova, Padova, Italy; ^9^Clinical Neuropsychology Unit, Hospital of the Sisters of Charity, Ried, Austria; ^10^Division of Neurosciences, University of Liverpool, Liverpool, United Kingdom; ^11^Department of Psychology, Linnaeus University, Växjö, Sweden; ^12^Department of Psychology, University of Oslo, Oslo, Norway; ^13^Department of Neurology, Akershus University Hospital, Lørenskog, Norway

**Keywords:** clinical neuropsychology, specialization, EuroPsy, training, competencies, standards, guidelines and recommendations

## Abstract

The prevalence and negative impact of brain disorders are increasing. Clinical Neuropsychology is a specialty dedicated to understanding brain-behavior relationships, applying such knowledge to the assessment of cognitive, affective, and behavioral functioning associated with brain disorders, and designing and implementing effective treatments. The need for services goes beyond neurological diseases and has increased in areas of neurodevelopmental and psychiatric conditions, among others. In Europe, a great deal of variability exists in the education and training of Clinical Neuropsychologists. Training models include master’s programs, continuing education courses, doctoral programs, and/or post-doctoral specialization depending on the country, with no common framework of requirements, although patients’ needs demand equal competencies across Europe. In the past 5 years, the Standing Committee on Clinical Neuropsychology of the European Federation of Psychologists’ Association has conducted a series of surveys and interviews with experts in the field representing 30 European countries. The information, along with information from the existing literature, is used in presenting an overview of current and relevant topics related to policy and guidelines in the training and competencies in Clinical Neuropsychology. An option for the way forward is the EuroPsy Specialist Certificate, which is currently offered in Work and Organizational Psychology, and in psychotherapy. It builds upon the basic certificate and complements national standards without overriding them. General principles can be found that can set the basis for a common, solid, and comprehensive specialty education/training, sharpening the Neuropsychologists’ competencies across Europe. The requirements in Clinical Neuropsychology should be comparable to those for the existing specialty areas in the EuroPsy model. Despite the perceived challenges, developing a specialist certificate appears a step forward for the development of Clinical Neuropsychology. Recommendations are proposed toward a shared framework of competencies by the means of a common level of education/training for the professionals in Europe. Benchmarking training standards and competencies across Europe has the potential of providing protection against unqualified and ethically questionable practice, creating transparency, raising the general European standard, and promoting mobility of both Clinical Neuropsychologists and patients in Europe, for the benefit of the professional field and the population.

## Introduction

According to the brief definition by the American Psychological Association:

*“Clinical Neuropsychology is a specialty field within Clinical Psychology, dedicated to understanding the relationships between brain and behavior, particularly as these relationships can be applied to the diagnosis of brain disorder, assessment of cognitive and behavioral functioning and the design of effective treatment*.”^1^

Clinical Neuropsychology in Europe has its roots in psychology, particularly experimental psychology, but also in neurology, psychiatry, and functional anatomy. The early milestones include the first chair of neurology created for Jean-Martin Charcot at the Salpêtrière in Paris France in 1882, the first descriptions of the localization of expressive speech by Paul Broca in 1861 and receptive speech by Carl Wernicke in 1874, and the clinico-pathological findings of Alois Alzheimer in 1906 (McHenry, 1969; Eling, 2016; Derouesné and Poirier, 2018). In 1879, Wilhelm Wundt (1832–1920), a physician, physiologist, and the first to call himself a psychologist, built a laboratory of experimental psychology in Leipzig, Germany. This is considered to mark the birth of psychology as an independent field of study. Wundt also contributed directly to neuropsychology by participating in the discussion of the theory of localization of function, and by developing theories of attention and cognitive control (Fahrenberg, 2015). Among the first to apply methods derived from experimental psychology to brain injury patients in Europe were Kurt Goldstein (1878–1965), a professor of neurology, and Adhémar Gelb (1887–1936), a psychologist who worked with him at the *Institut zur Erforschung der Folgeerscheinungen von Hirnverletzungen* (Institute for Research on the Consequences of Brain Injury) in Frankfurt am Main, Germany (Eling, 2016). Following the Second World War, psychologists across Europe were asked to evaluate the effects of penetrating head wounds in returning war veterans, which boosted the development of neuropsychology from experimental science to clinical specialty on a larger scale (Collins, 2016; Hokkanen et al., 2016).

Despite current evidence of the growing necessity for qualified health services regarding cognitive, affective, and behavioral consequences of long-term neurological conditions and psychopathological conditions, the training of specialists in Clinical Neuropsychology throughout the world, and even in Europe, is remarkably uneven. Only a minority of European countries, also in high-income regions, provide systematic high-level specialist training in Clinical Neuropsychology, as required for diagnosis and treatment of disorders described in the international classifications of diseases, namely ICD-10 and DSM V (Grote and Novitski, 2016; Hessen et al., 2018; Hokkanen et al., 2019). Both in France and in Spain, one of the top perceived barriers to the development of neuropsychology was reported to be the lack of clinical and academic training opportunities (Olabarrieta-Landa et al., 2016; Lopes et al., 2019). A consequence of this is that the general public in European countries receives neuropsychological care and treatment by professionals with unequal competencies and abilities to diagnose and treat their clinical conditions. In 2019, European Federation of Psychologists’ Association (EFPA) conducted a survey on the routes of specialization on all fields of psychology in Europe and while finding neuropsychology to be one of the most common areas of specialty, it also confirmed the heterogeneity of the existing training models (Dias Neto et al., 2020).

In 2015, EFPA established a Standing Committee on Clinical Neuropsychology to analyze the situation of the field of Clinical Neuropsychology in Europe and to make recommendations on how the specialization training in neuropsychology can and/or should be developed in the future.

The aims of the present paper are three-fold: (1) To discuss contemporary Clinical Neuropsychology, and to describe the professional situation of the field in Europe, (2) To describe the current specialization training models based on information collected by the EFPA Standing Committee on Clinical Neuropsychology, and contrasting it with the current EuroPsy Specialist Certificate model available for other areas in psychology, and (3) To make recommendations regarding benchmarking the training standards and competencies. We suggest that the EuroPsy Specialist Certificate model could play a critical role in ensuring the safety and care of the patients across Europe.

### The Role of Clinical Neuropsychology in Major Health Disorders

In considering the role of Clinical Neuropsychologists within the society, it is important to understand the diverse work areas of the practitioners in the field. The prevalence and health impact of brain disorders e.g., traumatic brain injury (TBI), stroke, epilepsy, motor neuron disease etc., are substantial. A study conducted in 2003 by the European Brain Council found that disorders of the brain were the largest contributor to the total morbidity burden in Europe, accounting for 35% of all disease burden (Olesen and Leonardi, 2003). The economic costs of disorders affecting the brain are large, constituting 24% of the total direct healthcare expenditure in Europe in 2010 (Gustavsson et al., 2011; Olesen et al., 2012). Notably, this study expands beyond the typical neurological disorders and acknowledges the impact of for example psychotic disorders, anxiety disorders, mood disorders, and neurodevelopmental disorders in childhood and adolescence (Olesen et al., 2012). The World Health Organization in their report estimated that while the neurological disorders they considered in 2005 globally contributed to 92 million disability-adjusted life years (DALYs), the burden is projected to increase to 103 million in 2030 which is approximately a 12% increase (World Health Organization, 2006).

The cognitive, affective, and behavioral disturbances related to brain disorders are a significant cause of disability, have a negative influence on functional outcomes, impact personal, professional, and social lives and significantly impair the quality of life of those affected and their family members (see Feigin et al., 2019). With the increasing prevalence of these disorders (World Health Organization, 2006; Feigin et al., 2019), the demand for health professionals with expertise both in assessment and treatment of these patients has also increased. Among those working in this field, the extensive training and core competencies of Clinical Neuropsychologists make them well suited to respond to the demand (Lezak et al., 2012; Hessen et al., 2018).

Clinical Neuropsychologists can help obtain important information for diagnostic criteria in order to differentiate between various clinical phenotypes of mental, neurological, or neurodevelopmental disorders, as well as evaluate their functional outcomes. In many countries, neuropsychological assessment has a role in quantifying and understanding deficits or impairments for the purpose of disability insurance and other legal implications. Neuropsychological assessment can also pinpoint the type of intervention needed, suggest the required amount of relevant rehabilitation or therapies, and evaluate the progress and efficacy of rehabilitation with objective measures of mental functioning (Lezak et al., 2012). This information is vital in evaluating the abilities of returning to work and maintenance or improvement of independence in daily life and social activities. A recent critical review found evidence of the incremental value of neuropsychological assessment e.g., in the care of persons with mild cognitive impairment/dementia, TBI, stroke, epilepsy, multiple sclerosis, and attention deficit hyperactivity disorder (ADHD; Donders, 2020). Based on the review, participation in neuropsychological evaluations was also associated with cost savings.

Similarly, Clinical Neuropsychologists can provide the rehabilitation services to, for instance, survivors of stroke, TBI, other forms of acquired brain injury or to those suffering from developmental deficits, such as dyslexia or autism. There are numerous studies indicating the evidence-based efficacy of neuropsychological interventions, such as those reviewed by Rohling et al. (2009), Cicerone et al. (2011, 2019), van Heugten et al. (2012), and Langenbahn et al. (2013). Moreover, Clinical Neuropsychologists can contribute to public health by educating people on how to best improve and maintain brain health during the entire lifespan, and they can also increase public awareness about neurocognitive or neuroaffective disorders and brain-behavior relationships.

Qualified assessment of neuropsychological function is now a requirement both for a diagnosis and for identifying potential functional disabilities due to different conditions affecting the brain. For example, current consensus requires identifying specific patterns of neuropsychological deficits in order to diagnose mild cognitive impairment in early stages of possible neurodegenerative diseases like Alzheimer’s disease (Albert et al., 2011) or Parkinson’s disease (Litvan et al., 2011). Importantly, neuropsychological assessment has proven valuable in the diagnosis and prediction of outcomes in elderly individuals who are at risk for MCI and/or progression to dementia above and beyond neuroimaging or biomarkers (Donders, 2020).

With respect to multiple sclerosis, cognitive symptoms were described in the first known article that addressed its clinical and pathological characteristics, written by Jean Martin Charcot (1877). In more recent years, two studies by Rao and colleagues (Rao et al., 1991a,b) revealed that cognitive and behavioral symptoms both were frequent and among the most disabling symptoms in many persons with multiple sclerosis. Further research has confirmed that neuropsychological symptoms may be the most disabling symptoms reported in multiple sclerosis (Stuifbergen et al., 2012) and thus important to detect and address. The increment value of neuropsychological variables has been found especially in predicting outcomes after multiple sclerosis (Donders, 2020).

Epilepsy, one of the most frequently diagnosed neurological conditions, is defined by the International League Against Epilepsy and the International Bureau for Epilepsy as “a disorder of the brain characterized by an enduring predisposition to generate epileptic seizures and by the neurobiological, cognitive, psychological, and social consequences of this condition” (Fisher et al., 2005). This definition does not only require the occurrence of epileptic seizures, but also the assessment of its impact on cognitive and psychological functions (McCagh et al., 2009).

Traumatic brain injury is another major health condition where Clinical Neuropsychologists have been playing a critical role in research, assessment, treatment, and rehabilitation over the last several decades (Yeates et al., 2017). Moreover, neuropsychological assessment, quite often, provides the only objective functional measure of mild traumatic brain injury, which represents the vast majority of traumatic brain injury cases. Thus, neuropsychological assessment ensures the best understanding of the cognitive, affective, and behavioral consequences of mild traumatic brain injury (Zink, 2001), allotting neuropsychology a key role in planning rehabilitation and treatment following TBI. Neuropsychological test variables add uniquely to the prediction of outcomes after TBI, in both children and adults (Donders, 2020).

Among neurodevelopmental disorders, ADHD affects people across the lifespan with a prevalence rate of 3–5% in childhood, 1.4–3.6% in adulthood, and 2.8–4.2% in persons over 60 years of age (Kooij et al., 2019). In the United States, 11% of all children of 4–17 years have ever had a diagnosis for ADHD (Visser et al., 2014). For those affected, neuropsychological assessment provides a precise description of the cognitive problems and offers specific information for individualized treatment planning (Lange et al., 2014). Early neuropsychological assessment may also be of incremental benefit in the prediction of the development of ADHD and the associated outcomes (Donders, 2020). ADHD has risen to be the most common condition seen by Nordic neuropsychologists (Norup et al., 2017); among the French neuropsychologists, ADHD and learning disabilities were at the top third and fourth place in diagnostic groups of clients for assessment and rehabilitation (Lopes et al., 2019).

In addition to neurological and neurodevelopmental disorders, neuropsychological dysfunctions also appear as core features of some psychiatric disorders. For example, schizophrenia is associated with moderate to severe deficits across several cognitive domains, including attention, working memory, verbal learning and memory, and executive functions (Elvevag and Goldberg, 2000; Kremen et al., 2000; Sheffield et al., 2018). Similar findings are also evident in depressive psychosis. Studies have consistently found that these deficits in the case of schizophrenia pre-date the onset of frank psychosis and are stable throughout the course of the illness in most patients (Sheffield et al., 2018). With the recognition that neuropsychological deficits are consistently the best predictor of functional outcomes across outcome domains and patient samples, the focus on cognition has increased dramatically (Bowie and Harvey, 2006). Acknowledging the importance of neuropsychological functioning in schizophrenia, the DSM-5 (American Psychiatric Association, 2010) recommends obtaining a formal neuropsychological assessment in individuals with psychosis (Elvevag and Goldberg, 2000; Kremen et al., 2000; Bowie and Harvey, 2006).

### Clinical Neuropsychology as a Specialist Profession

The development of professional Clinical Neuropsychology has mainly taken place in high income regions of European countries, Australia, and North America, with well-developed health care systems. This is reflected both by the number of scientific publications that come from these areas of the world and by the growth of Clinical Neuropsychology in comparison to other psychological disciplines. One clear example of this is evident in the United States, where the Division of Clinical Neuropsychology now is the largest of 55 divisions of the American Psychological Association.^2^ In addition, the board certification in Clinical Neuropsychology has grown faster over the last few years than any of the other 13 specialties under the umbrella of the American Board of Professional Psychology, including Clinical Psychology (Hessen et al., 2018).

After the Second World War, professional development in European countries progressed but not linearly. As stated by Collins (2016), “separate neuropsychology developed when psychologists could offer techniques they could call their own, most notably psychometric tests, when psychology itself became an established academic discipline and set of professional practices, when psychologists began to occupy posts that brought them into regular contact with brain-damaged patients, and when psychologists began to develop models that were adequate for explaining both normal and disordered function.” In the United Kingdom, these steps were taken in the second half and especially in the last quarter of the twentieth century (Collins, 2016). In Germany, progress was hindered by the National Socialism regime and the forced emigration of notable researchers, such as Kurt Goldstein and Hans-Lukas Teuber, who took refuge in the United States (Eling, 2016). In several European countries, the move from research labs to clinical applications, and from the rehabilitation of war veterans to wider civilian health care has taken its time (Boller et al., 2016; Collins, 2016; Eling, 2016; Hokkanen et al., 2016).

The evolution of the professional expertise in Norway can serve as one example. In 1987, the specialty of Clinical Neuropsychology was separated from the general specialty in Clinical Psychology. At that time, the service provided by neuropsychology was insignificant and with hardly any impact on public health care. Today, the service provided by specialists in Clinical Neuropsychology has become a requirement in the diagnostic processes as well as in treatment planning and implementation, within all relevant aspects of specialist health care (Hessen et al., 2016). Thus, positions for specialists in Clinical Neuropsychology exist within major hospital departments, namely, in child and adult psychiatry, pediatrics, neurology, rehabilitation, and geriatric health care. Clinical Neuropsychology has emerged and established itself as a new required health service over the last 30 years.

A different example of the development of Clinical Neuropsychology comes from France. Although the history of French neuropsychology is long, beginning with Broca in 1861, its evolution from being part of neurology to its recognition as an autonomous discipline has been slow (Derouesné and Poirier, 2018). The first master’s degree specializing in neuropsychology was opened in 1992, yet an association aimed at promoting professional neuropsychology in France (*Organisation Française des Psychologues spécialisés en Neuropsychologie* – the Organization of French Neuropsychologists) was only created in 2014 (Lopes et al., 2019). Additionally, the allocation of public funding in France still tends to put psychologists at a disadvantage in relation to other professions working in the field (Derouesné and Poirier, 2018).

### Estimated Numbers of Practitioners in Europe

Estimating the current number of practitioners across Europe is challenging. Legal regulations usually do not exist, and the definition of a Clinical Neuropsychologist varies from country to country (Hokkanen et al., 2019). A survey conducted by the EFPA Standing Committee on Clinical Neuropsychology consisting of information provided by representatives of national psychological associations and/or neuropsychological societies in Europe suggested a total number of active practitioners within Clinical Neuropsychology in Europe being 13,367 (Hokkanen et al., 2019). Relative to the population in Europe, it amounts to one practitioner for 53,494 individuals. There is a great deal of variation between countries, however, ranging from 1 per 10,455 inhabitants in Denmark to 1 per 1,995,250 in Turkey (Hokkanen et al., 2019). The numbers presented do not indicate that all practitioners have equal education, training, competencies, and job descriptions. No other similar studies from across Europe can be found for comparison. Prior estimates in individual European countries included in a global survey have suggested 1 per 19,231 for Finland and 1 per 32,000 for Spain (Grote and Novitski, 2016). The ratio of Neuropsychologists to Clinical Psychologists in the global survey of 14 countries suggested a mean ratio of 1:29 (Grote and Novitski, 2016).

Another way of estimating the number of Clinical Neuropsychologists comes from looking at the size of the membership in national neuropsychological societies in Europe. There are 23 societies that are members of the Federation of the European Societies of Neuropsychology (FESN). Some have less than 50 individual members, many between 200 and 400 members, some over 1,500. Many of the societies accept members representing different educational backgrounds such as physicians and speech therapists, in addition to psychologists. Membership is often not restricted to those with specialization training and the numbers may therefore also reflect the general interest in neuropsychology among students and other practitioners. Some countries also have several societies representing different geographical regions, different languages, or different professional focus and goals. Some include members working in academic or research settings, in addition to clinical practitioners. As a result, the estimates on the numbers of practitioners differ. For instance, the estimated number of neuropsychologists in France (5,000) refers to the number of graduates with a Master’s degree in Neuropsychology reported by the French National Association of Neuropsychologists (Hokkanen et al., 2019; Lopes et al., 2019). At the same time, the number of members of the Société de Neuropsychologie de Langue Française (French Speaking Neuropsychological Society) who are also members of the FESN, is 220. In a recent report from France, the number or members in the Organisation Française des Psychologues Spécialisés en Neuropsychologie (Organization of French Neuropsychologists) is 500, and the number of those who self-identified as psychologists or other health professionals working in the field of neuropsychology responding to the survey was 800 (Lopes et al., 2019).

## Specialist Education Options and Implications

As stated in the previous section, the work area of Clinical Neuropsychology is wide and diversifying, involving a high number of different etiologies and treatment strategies. The required competencies reflect this fact. The development of the profession in the field is at different stages in different countries, as seen in the few examples above.

### Current Training Models

In Europe, a great deal of variability currently exists among countries with respect to the models of training to become a Clinical Neuropsychologist. Based on the survey conducted by the EFPA Standing Committee on Clinical Neuropsychology (Hokkanen et al., 2019) there are training models based on master’s programs, continuing education courses, doctoral programs, and post-doctoral specialization. Table 1 lists training models in European countries as described in the 2017 survey.

**Table 1:** Features in the specialization and training of Clinical Neuropsychologists in Europe (total number of responding countries was 30) based on the data in Hokkanen et al. (2019).

Feature	Frequencies	Length (mean ± standard deviation, minimum – maximum)
Current model for specialization	Pre-planned program 11 (37%) Individual courses 6 (20%) Flexible routes 3 (10%) No commonly agreed model 10 (33%)	
Education required to practice clinical neuropsychology	Bachelor’s level 2 (7%) Master’s level 14 (48%) Doctoral level 2 (7%) Other 9 (28%) Not defined 3 (10%)	6.7 ± 2.1 years^1^ range 3.5–12 years
Specialist education after the master’s level		32.9 ± 13.9 months range 12–60 months
Practical training	Required 21 (70%) Not required 5 (17%) Missing data 4 (13%)	23.9 ± 20.7 months range 3–60 months
Providers of the training programs^2^	University 11 (55%) National ministry or authority 4 (20%) National psychological association 5 (25%) National neuropsychological society 6 (30%) Another public organization 0 Private / commercial training institute 4 (20%)	
Providers of individual courses^2^	University 11 (55%) National ministry or authority 2 (10%) National psychological association 8 (40%) National neuropsychological society 9 (45%) Another public organization 3 (15%) Private / commercial training institute 5 (25%)	

1 Including the years required on top of the academic degree if applicable.

2 Countries with a model in place (*n* = 20).

In the United States, specialist education and training in Clinical Neuropsychology has been defined in *The Houston Conference on Specialty Education and Training in Clinical Neuropsychology* policy statement (Hannay et al., 1998). The statement is grounded on the view that the training of the specialist in Clinical Neuropsychology must be based on the scientist-practitioner model (Belar and Perry, 1992), and it may lead to a primarily practice, primarily academic, or a combined career. Within the Houston model, the specialization in Clinical Neuropsychology begins at the doctoral level including an internship period and continues in a post-doctoral residency or fellowship training program (Hannay et al., 1998).

In Europe, Doctoral level education for Clinical Neuropsychologists is a requirement only in the United Kingdom and Ireland. In these countries, practicing Clinical Psychology also requires a doctorate. Psychology education in the United Kingdom, however, does not follow the three-cycle Bologna model^3^ where a minimum of a 3-year bachelor’s degree is followed by a 2-year master’s degree before entering doctoral training is possible. In the United Kingdom, doctoral training can begin after the bachelor’s degree. Also, a clinically oriented Doctor of Psychology (D Clin Psychol) is available in the United Kingdom in addition to the more research-intensive Doctor of Philosophy (PhD) degree. Similar dual doctorate routes are available also in Australia and the United States, but generally in Europe only a PhD is available. Within the specialist education in Clinical Neuropsychology, the requirements related to research skills vary in the seven countries reviewed in Hessen et al. (2018): In addition to Australia and the United States, Italy also requires a dissertation, but in Italy, this does not lead to a doctorate. In the United Kingdom, an empirical research study is required that contributes to a doctorate. In Finland, the Netherlands, and Norway, students produce one or two scientific papers as part of their specialist education, either in the form of systematic literature reviews or empirical studies, published or not (Hessen et al., 2018).

### Competencies in Clinical Neuropsychology

The competency approach became prominent in medical training in early 2000 (Leung, 2002; Williams et al., 2010) with various international initiatives promoting competency-based training and assessment (e.g., The European Union of Medical Specialists). Core competencies for professional practice were also specified internationally for psychology by the means of the International Declaration of Core Competences in Professional Psychology.^4^ In Europe, the general competencies necessary for practicing psychology were further developed within the EuroPsy model (Lunt et al., 2015).

Competencies for each specialist area of psychology need to be separately identified, however. In response to this need, core competencies for entry-level Clinical Neuropsychologists have been delineated in the United States (Rey-Casserly et al., 2012) and approved by the American Board of Professional Psychology/American Board of Clinical Neuropsychology and the Council of Specialties in Professional Psychology (COSPP).^5^ Practicum guidelines and methodology for competency-based evaluation of Clinical Neuropsychology trainees were also established (Nelson et al., 2015). Likewise, in the United Kingdom a competency framework in Clinical Neuropsychology was published by the British Psychological Society (Division of Neuropsychology, 2012).

The framework of COSPP delineates the competencies necessary for entering the professional practice in health care (the so-called entry-level competencies). They are divided into foundational competencies (knowledge-based elements necessary across all of the neuropsychologist’s functional domains), functional competencies (knowledge- and skill-based elements describing particular aspects of practice), and additional competencies (relevant in specific advanced areas of practice). Each competency area is described in terms of several individual subcompetencies that are necessary for a successful practice.

In the Assessment domain, examples of the knowledge-based subcompetencies (total of 10) include the knowledge of the neuropsychology of behavior (involving information processing theories, cognitive/affective neuroscience, social neuroscience, cultural neuroscience, and behavioral neurology); the knowledge of patterns of behavioral, cognitive, and emotional impairments associated with neurological and related diseases and conditions that affect brain structure and functioning; the knowledge of patterns of behavioral, cognitive, and emotional impairments associated with psychiatric disorders; and the knowledge of theories and methods of measurement and psychometrics relevant to cognitive abilities, social and emotional functioning, and brain-behavior relationships. Examples of skill-based subcompetencies in the Assessment domain (total of nine) include the ability to analyze and clarify referral questions based on the context, professional roles, and the patient/examinee presentation; the ability to gather information key to addressing the referral question, including interview(s), targeted behavioral observations, and review of records; the ability to appropriately select tests, measures, and other information sources consistent with best evidence and specific context of assessment, including assessment of performance and symptom validity, if relevant; the ability to interpret assessment results, with formation of an integrated conceptualization that draws from all relevant information sources (e.g., interview, test results, behavioral observations, and records); and the ability to demonstrate written communication skills in the production of integrated neuropsychological assessment reports.

Similarly, the Intervention domain includes five knowledge-based and seven applied competencies, and the Consultation domain includes three knowledge-based, seven applied competencies. The additional competency domains include research, teaching and supervision, management-administration, and advocacy, each with their own knowledge-based and applied subcompetencies. See the COSPP framework^6^ for the full list.

Recently, these entry-level competencies were evaluated in a global comparison between individual training programs in Australia, Finland, Italy, Netherlands, Norway, United Kingdom, and United States, and the agreement for the required competencies was quite high (Hessen et al., 2018). The assessment domain was covered very similarly in all countries included, but small variation existed in the amount of focus on the fields of neurochemistry, neuropsychopharmacology, and neuroendocrinology, and also on the amount of emphasis on addressing issues related to specific populations (Hessen et al., 2018). Within the intervention domain, both the knowledge base and the application are, with few exceptions, covered similarly.

### EuroPsy Specialist Certificate Model

The European EuroPsy model^7^ offers a framework that can be used in developing basic and specialist education and training. The general aims of EuroPsy are (1) protection of consumers and citizens in Europe by providing quality assurance and protection against unqualified and ethically questionable practice, (2) promotion of the availability of adequate psychological services across Europe by creating transparency and raising standards, and (3) promoting the mobility of psychologists (and clients) in Europe (Lunt et al., 2015).

Twenty-four countries in Europe have adopted the EuroPsy Basic Certificate, which includes 6 years of professional education in Psychology (5 years academic and 1-year supervised practice), declaration of ethical behavior, and an obligation for continuing professional development. The general competencies of psychologists are also outlined within the framework. The workload is described in terms of the European Credit Transfer and Accumulation System (ECTS) where one academic year is equivalent of 60 ECTS. Despite differences in defining the exact curriculum and requirements for psychology practitioners, all EFPA member associations agree on the general structure and competencies it involves (Lunt et al., 2015). Importantly, the EuroPsy is a European qualification that complements but does not override national standards.

The EuroPsy Specialist Certificate builds upon the basic certificate and is currently offered in Psychotherapy and Work and Organizational Psychology (Lunt et al., 2015; Dias Neto et al., 2020). The requirements for the basic EuroPsy certificate need to be met in order to apply for the Specialist Certificate, but it is possible to apply for both certificates at the same time. Table 2 describes the current requirements in the two available specialist areas, both involving 90 ECTS of training and 3 years of supervised practice. European countries vary in the degree to which they have developed specialization.^8^ The EuroPsy Specialist Certificate in Psychotherapy is currently available in Finland, Russia, Spain, and Turkey. The EuroPsy Specialist Certificate in Work and Organizational Psychology is available in Finland, Norway, and Spain.

**Table 2:** Description of the requirements in the currently available specialist certificates within the EuroPsy framework, Psychotherapy and Work and Organizational Psychology.

Requirement	Psychotherapy	Work and Organizational Psychology
Education	90 ECTS, of which 400 h theory	90 ECTS (2,400 h)^*^, of which 60 courses and 30 applied research/assessment/intervention
Content	Vary with curriculum and/or learning trajectory	Specialist curriculum framework
Supervised practice	3 years, 500 h of work supervised	3 years, 400 h/year supervised, “coached professional practice”
Supervision	150 h (50 h/y)	150 h (50 h/y)
Competencies to be demonstrated	A list is being developed.	Those defined in EuroPsy regulations, applied to Work and Organizational Psychology specialized level
Reflection is required on	Psychologists as Psychotherapists Inquiry and communication Psychotherapy practice and understanding Personal and professional development Ethical and competent practice Further professional development	
Competence development	Implied in Psychotherapy training in one or more Psychotherapy methods	Based on an explicit system of Competence Development
Continued professional development	Being developed	4 ECTS (100 h) at the moment of application, included in the 90 ECTS
Additional requirement	100 h personal therapy	

* Early specialization countries: 60 ECTS after basic EuroPsy ECTS, European Credit Transfer and Accumulation System; h, hours.

The implementation of the certificate system is outlined in the EuroPsy Regulations.^9^ Within EFPA, there is a European Awarding Committee (EAC) for the basic certificate and a European Specialist Awarding committee (SEAC) for the specialist certificates. The authority to award the certificates has been delegated to national level, to National Awarding committees (NACs) and Specialist National Awarding committees (S-NACs) in countries that have adopted the EuroPsy model. The EAC and the SEACs supervise the proper implementation of the regulations, ensure that the national bodies are interpreting the European standards in a similar way, and coordinate the work of the NACs and S-NACS in many ways. The national committees report to and submit all their procedures for approval to the European level committees. This ensures the compliance with the common standards.

### Challenges in the Common EuroPsy Model

The EuroPsy Specialist Certificate can be seen as an instrument for benchmarking common standards in training and competencies. There are challenges, however, that can occur on several levels. One is related to agreeing on the model and the standards. Second is related to the interest in actual use of the certificates. Third is the assumed impact of the certification system.

Identifying common minimum requirements for a qualification in Clinical Neuropsychology would be helpful for developing national training programs and would reduce the heterogeneity in different programs and practices. The standards may appear too high in relation to the current situation in some countries, however. If the development is still in progress, the recommendations should be seen as aspirational and not condemnatory. The differing educational models, such as early specialization, also need to be taken into account in defining the requirements (Dias Neto et al., 2020). Another concern applies to countries with already established high standards. If the commonly agreed model includes lower standards, it may create a situation where training abroad results in certification that would not have been approved in the home country. This risk needs to be considered. Countries with established regulations and requirements, if higher than the proposed model, will probably want to keep the original requirements in place. Within the EuroPsy Specialist Certification system this is possible, as this certification does not supersede national standards.

The Specialist Certificate builds upon the basic certificate. The basic EuroPsy are currently offered in 24 European countries, but the application rates vary. In Portugal, most of the about 19,000 effective members of the *Ordem dos Psicólogos Portugueses* (Portuguese Psychologists’ Association) applied for and obtained the basic EuroPsy. However, in France among 70,000 psychologists, less than 150 professionals have obtained the EuroPsy basic certification. It is voluntary, so practitioners will consider the need based on their personal situation. The 1-year supervised clinical practice required for EuroPsy is not included in the basic training in all countries, which means it must be obtained separately. There may be difficulties in finding a job where supervision can take place. In France, young graduates need time to find their first job, often in part-time and with a short fixed-term contract with low salaries. There are also financial costs involved in the certificate application process that may be covered by the national association in some countries but left to the practitioners themselves in others. Paying for an optional certificate may not be a priority. Still, a proposal of a Specialist Certificate involves strategic aims for benchmarking European standards which will be beneficial regardless of the number of practitioners applying for the certificate.

Although the Basic or Specialist Certificates are not mandatory to practice psychology, there are relevant incentives to apply for them: (a) there is a register/directory of EuroPsy certified psychologists with national listings that can be consulted by those seeking the services of qualified psychologists,^10^ (b) through the EuroPsy, EFPA encourages psychologists to obtain continuing and specialized training, (c) obtaining a Specialist Certificate provides professional enrichment, valorization of training, delineation of specific contexts of practice, and can be a process for rewarding merit and competency (Dias Neto et al., 2020), and (d) the recognition of a specialization at a European level fosters mobility and sharing of knowledge between nations (Dias Neto et al., 2020).

The Specialist Certificate and the EuroPsy is believed to have implications on the system’s level (protection of consumers by raising standards) as well as the individual level (promoting the mobility) of the practitioner (Lunt et al., 2015). In countries with established standards and regulation already in place, the new certification may not offer much incremental value within health care. Overall, the picture of psychology specialization, however, is still in its infancy (Dias Neto et al., 2020). The best means for both protecting the public in need of neuropsychological services and developing professional practice are still under debate, and certification may not be the only potential model. For individual health professionals moving across borders, there are considerable language and legal restrictions that may hinder mobility even for the holders of the certificate. For trainees, it might still open new opportunities and promote knowledge transfer.

The impact of common standards on the level of care within society is linked to the licensure policies. A certificate might not be helpful if the title or the practice of Clinical Neuropsychology is not protected by law. In Europe, most countries regulate practicing clinical psychology but not neuropsychology (Hokkanen et al., 2019). After obtaining licensure for clinical psychology, adequate training and competence in other specialist fields of psychology is demonstrated separately and the authorities evaluating the qualifications differ (Dias Neto et al., 2020). In the United Kingdom for example, the Qualification in Clinical Neuropsychology involves a clinical portfolio and an oral examination. This resembles the American Board of Professional Psychology Clinical Neuropsychology certification exams. The qualification, however, is not legally required for practicing neuropsychology, and the patients seeking services may not be aware of such qualifications. EuroPsy Certificates do not override national laws or regulations, and do not provide licensures to practice psychology in any particular country. They are, however, a common framework to recognize qualifications across Europe. If a European standard for Clinical Neuropsychology education and training existed, it would serve as a tool for advocating the need for separate licensure also for Clinical Neuropsychology in the future. Also, informing the public would be easier.

## Recommendations and Discussion

The statistics on the major health disorders where Clinical Neuropsychology is relevant support the need for expert and competent neuropsychological services within health care. There is a clear discrepancy between the number of trained Clinical Neuropsychologists vs. the demand for those services across long term neurological conditions. The European Brain Council called for political action, and quantitatively and qualitatively improved teaching at medical schools and other health-related educational programs, including psychological treatments (Gustavsson et al., 2011). The differences in means and timings for acquiring a specialty in psychology have been suggested to weaken the profession of psychology (Dias Neto et al., 2020). The education and training in Clinical Neuropsychology need to rise to the challenge.

The general framework of specialist education and training in Clinical Neuropsychology can be based on a few grounding values. In their paper on the training models of Clinical Neuropsychologists in Europe, the Standing Committee on Clinical Neuropsychology described four principles that could potentially be used as bases in establishing the common requirements for specialist education and training (Hokkanen et al., 2019). These are: (1) Commencing the Specialist education in Clinical Neuropsychology should be preceded by at least 5 years of higher education in psychology culminating in a master’s degree (or equivalent) and a minimum of 1-year clinical practice, (2) The core elements of the specialist education should include theoretical study, practical training with supervision, and research experience, (3) The theoretical studies, whether in the form of a program or a combination of separate courses, should be accredited by a national authority, and (4) The length, depth, and breadth of the different elements within the specialist education must be sufficient to allow for the accumulation of the advanced competencies necessary for successful entry into the profession. Achieving these competencies typically requires several years of specialization in Clinical Neuropsychology. As these principles are in accordance with the EuroPsy model and the existing Specialist Certifications, two recommendations are in order.

Recommendation 1: Commence the process to develop a EuroPsy Specialist Certificate in Clinical Neuropsychology.

Recommendation 2: Review the competency areas in the European framework in order to find a common ground for the learning objectives.

The number of countries currently offering EuroPsy Specialist Certificates in other areas of psychology is gradually increasing. While progress may be slow, the overall development of these fields has been greatly enhanced by the communication among professionals across Europe. Similar development is welcome and urgent in Clinical Neuropsychology as well. For countries with existing high standards for the education and training in Clinical Neuropsychology, a common framework will offer further consolidation of the field without losing their national regulatory power and ensure that incoming Clinical Neuropsychologists are better prepared. For countries in which Clinical Neuropsychology is still developing, the framework will provide aspirational standards in education and professional practice. This will help to bridge the differences among European countries regarding training and required competencies for Clinical Neuropsychologists, and ultimately pave the way for universal higher quality practices in the delivery of Clinical Neuropsychological services across Europe for the benefit of the patients.

## Author Contributions

LH wrote the first draft of the manuscript and finalized it for submission. FB, AP, MC, MK, NV, EK, SM, SL, GB, BP, and EH had intellectual contributions to the content. All agreed to the submitted version of the publication. All authors contributed to the article and approved the submitted version.

### Conflict of Interest

The authors declare that the research was conducted in the absence of any commercial or financial relationships that could be construed as a potential conflict of interest.

## Footnotes

^1^https://www.apa.org/ed/graduate/specialize/neuropsychology

^2^http://www.apa.org/about/division/officers/services/profiles.aspx

^3^http://www.ehea.info/page-three-cycle-system

^4^www.asppb.net/news/297538/International-Project-on-Competence-in-Psychology-IPCP

^5^http://cospp.org/specialties/clinical-neuropsychology

^6^http://cospp.org/specialties/clinical-neuropsychology

^7^http://www.europsy.eu/

^8^www.europsy.eu/quality-and-standards/europsy-specialisation

^9^https://www.europsy.eu/_webdata/europsy_regulations_july_2019_moscow.pdf

^10^https://www.europsy.eu/search-psychologist
